# Improving policy, financing and delivery of postnatal care services

**DOI:** 10.2471/BLT.22.289440

**Published:** 2023-01-01

**Authors:** Etienne V Langlois, Teesta Dey, Domenico G Iaia, Emma Sacks

**Affiliations:** a Partnership for Maternal, Newborn & Child Health, World Health Organization, Avenue Appia 20, 1211 Geneva 27, Switzerland.; b Department of International Health, Johns Hopkins School of Public Health, Baltimore, United States of America.

Postnatal care has long been neglected within the essential package of services across the continuum of reproductive, maternal, newborn and child health.^1^ Yet, the highest burden of mortality and morbidity for mothers and infants occurs within the postnatal period and is largely preventable with access to high-quality and timely care.^2^ Increasing coverage of postnatal care for vulnerable populations is needed, including adolescent mothers, refugee families and other disadvantaged groups.

The postnatal period (6 weeks after childbirth) is a vital opportunity to improve maternal and newborn health through supporting healthy behaviours, facilitating breastfeeding, counselling about family planning options, and preventing and treating subsequent maternal complications and neonatal health issues.^3^ This period is also an opportunity to initiate early childhood development interventions, thus stimulating integration of care along the continuum of essential reproductive, maternal, newborn, child and adolescent health services.

Despite these facts, estimates of postnatal care coverage across 66 low- and middle-income countries show that over 15% of mothers and infants still do not receive any postnatal care.^2^ The COVID-19 pandemic response has exacerbated poor coverage. As of July 2022, sustained disruptions to postnatal care continue, with only 46 out of 84 countries assessed delivering essential postnatal care services at a pre-pandemic level.^2^

The World Health Organization has recently updated its recommendations on maternal and newborn care for a positive postnatal experience, providing the latest guidance on evidence-based interventions to inform policy and practice.^4^ The guidelines support recommendations that place women and newborns at the centre of care, including health workforce interventions such as task-sharing and midwifery-led continuity of care models.^4^

Investments in postnatal care are highly efficient and have lasting impacts on reducing preventable maternal and newborn mortality and morbidity, enhancing sexual and reproductive health and rights and promoting a life course approach to health and well-being. For example, promotion of breastfeeding can yield a return of about 35 United States dollars (US$) for each US$ 1 invested.^5^ Recent evidence shows that immunization starting in the postnatal period is among the most cost-effective interventions across the life course.^6^ High-quality family planning counselling during the postnatal period also generates significant savings for public health investments.^7^^,^^8^

Therefore, supporting the inclusion of postnatal care services in universal health coverage benefits packages is needed. Comprehensive and integrated postnatal care for mothers and infants should be integral to basic packages of primary health-care services. This integration would help mitigate the disruption to essential services in the face of outbreaks and crises, thus enhancing the resilience of maternal and newborn health care. These suggested strategies require upfront investments in human resources for health, access to health systems and support to quality of care.

Concerted action is required to improve both supply and demand for postnatal care. Stakeholders within and outside the health system have a key role to play to devise intersectoral strategies that enhance the coverage of postnatal care. Innovations including digital health education and communication are promising to increase the coverage of these services where access may be more limited.^4^ Efforts are also required for community engagement that supports behaviour change and uptake of postnatal care for mothers and infants.

Having a sufficient health workforce that is trained and supported to provide quality postnatal care is also crucial. Efforts are required to address the shortage of 900 000 midwives worldwide.^9^ Decision-makers should prioritize policies enhancing human resources for health and investments in pre-service and in-service training on the delivery of postnatal care.

Community health systems are critical to deliver these services, especially for vulnerable populations; therefore, community health workers and platforms need more support. Greater investments are needed to enhance quality of care and respectful care practices across health cadres, intrinsically linked to the uptake of postnatal care and continuity of maternal and newborn health.

Many countries do not have sufficient or accurate data monitoring systems in place to consistently collect information for postnatal care services, including birth notifications and registrations, and quality-of-care indicators. These data are critical to monitor and regulate services and hold stakeholders accountable to delivering essential and high-quality postnatal care in all settings. 

Unfortunately, postnatal care is largely undervalued by communities and often perceived as a low priority for health professionals; these perceptions negatively influence the uptake and coverage of care.^3^^,^^10^ Among various solutions, antenatal care contacts provide a strategic opportunity to promote the value and importance of care following childbirth. 

Now is the time to raise the bar on policy, financing and service delivery for essential postnatal care services. These efforts will be critical to accelerate progress and achieve the sustainable development goals related to maternal and newborn health by 2030.
